# Postmortem Investigation of Immunohistochemical Staining and Gross Description of Sarcomatoid Carcinoma of the Lung in a Patient With Extreme Leukemoid Reaction

**DOI:** 10.1177/2324709619860547

**Published:** 2019-07-05

**Authors:** Ali Ammar, Austin Ellis, Julia Hegert, Timothy W. Jones, Rumi Khan

**Affiliations:** 1 Orlando Health, Orlando, FL, USA; 2 Arnold Palmer Hospital for Children, Orlando, FL, USA

**Keywords:** sarcomatoid carcinoma, extreme leukemoid reaction, rare lung cancer, giant cell carcinoma

## Abstract

A 72-year-old male smoker was brought into the emergency department complaining of 4 months of progressive dyspnea and fatigue. Computed tomography angiogram of the lungs was negative for pulmonary embolism; however, a 10 cm right upper lobe mass and multiple bilateral pulmonary nodules were identified. While computed tomography scan of the head showed no lesions in the brain, there was osseous destruction of the right mandible. Records obtained from an outside hospital indicated that he had 2 prior biopsies of this lung mass that failed to show malignant cells. In addition, an outpatient positron emission tomography scan had shown increased tracer uptake in this mass as well as multiple nodules in the contralateral lung and in the left adrenal gland. This gentleman was admitted for sepsis and was started on broad-spectrum antibiotics. He continued to have respiratory compromise and required transfer to the intensive care unit for intubation and mechanical ventilation. Over the next 4 days, the patient progressed into septic shock requiring vasopressors and developed worsening respiratory failure. His white blood cell count continued to rise and peaked at 157 × 10^3^ cells/µL. The patient’s wife decided to proceed with comfort measures and the patient subsequently expired. Autopsy was consistent with sarcomatoid carcinoma, also known as giant cell carcinoma of the lung. Immunohistochemical staining was also performed, which identified several tumor markers as well as distant metastasis, hemorrhage, and multi-organ necrosis.

## Background

Sarcomatoid carcinoma of the lung, also known as giant cell carcinoma of the lung (GCCL), is a very rare and extremely aggressive cancer of the lung. It comprises only 0.3% to 4.7% of all lung malignancies.^1^ Despite poor outcomes, literature remains limited to case reports and only a few case series. GCCL has been observed more frequently in males than in females,^2,3^ and greater in smokers as compared with nonsmokers. The mean age of diagnosis is at 62.3 years.^3^ In 1991, a case series described the immunohistochemical markers and staining of 9 patients with GCCL.^4^ We rely heavily on immunohistochemical (IHC) for diagnosis of lung cancer. For example, cyotkeratin 5/6 and p63 are well-known markers of squamous cell carcinoma. Moreover, thyroid transcription factor 1 may be found in adenocarcinoma. Literature is clearly lacking in description of microscopic and macroscopic findings in GCCL. This case describes the clinical course and autopsy findings of an unfortunate patient diagnosed with GCCL.

## Case Presentation

We present the case of a 72-year-old male smoker presenting to the emergency department complaining of 4 months of progressive fatigue and dyspnea. He has a history of traumatic pneumothorax approximately 50 years ago during the Vietnam War and prior diagnosis of bladder cancer treated with cystoscopic resections and BCG (bacillus Calmette-Guérin) installations. More recently, he had 4 hospitalizations in the last 6 months for acute respiratory failure and was discovered to have an 8 cm mass in the right lung with multiple sites of suspected metastasis. Records of an outpatient positron emission tomography scan showed increased uptake in the left adrenal gland, contralateral lung, spleen, and multiple mediastinal lymph nodes. This lung mass was previously biopsied twice and failed to identify any malignancy; rather, these biopsies only showed cellular necrosis. Moreover, he was recently discharged after a 2-week-long hospitalization for hemorrhagic shock requiring multiple blood transfusions after a major bleed post-adrenal biopsy, which also failed to identify cytologic malignancy. During that hospitalization, bone marrow aspiration was performed, which was also nondiagnostic because it was a dry tap that failed to show any bone marrow cellularity.

On presentation to our emergency department, the patient was afebrile at 98.4°F, blood pressure was 125/66 mm Hg, heart rate 118 beats per minute, and oxygen saturation was 91% while on room air. On examination, patient appeared in moderate distress, tachypnic at 26 breaths per minute, there was no wheezing or crackles; however, there was decreased air entry bilaterally. White blood cell (WBC) count was noted to be extremely elevated at 69.3 × 10^3^/µL, platelets 95, and hemoglobin of 6.9. Computed tomography angiogram identified a 10 cm right upper lobe mass associated rib erosion, multiple masses and pulmonary nodules bilaterally, a hemorrhagic mass in the right adrenal gland measuring 8.5 × 5.5 × 4.5 cm, multiple small masses in the left adrenal gland, and splenomegaly measuring 19 cm. Computed tomography scan of the head and neck did not show any lesions in the brain; however, right mandibular osteonecrosis with presumed adjacent dental abscess were seen.

He was admitted and sepsis protocol was initiated. He was resuscitated with crystalloid fluids and given 2 units of packed red blood cells. He was treated with broad spectrum antibiotics to cover postobstructive pneumonia. Despite aggressive therapy, his WBC count continued to rise without any improvement of his respiratory distress. On day 2, repeat chest X-ray showed worsening infiltrates and bilateral pulmonary edema. Tachypnea worsened and the patient was placed on bi-level positive airway pressure. His respiratory failure progressed, and he required intubation with mechanical ventilation. On day 3, the patient experienced an episode of supraventricular tachycardia and required amiodarone in addition to diuresis and vasopressors for hemodynamic support. On day 4 of his hospitalization, the WBC peaked to 196.1 × 10^3^/µL. In light of his continued decline and poor prognosis, his wife expressed his wishes for comfort care only and for withdrawal of life support. He was extubated and was subsequently pronounced dead without having a definite diagnosis. Partial autopsy was performed, which provided light on his disorder.

**Figure 1.**
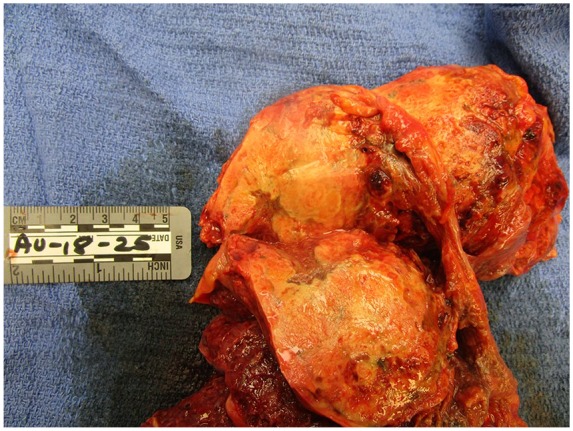
Right lung: large masses are visualized at the apex of the right lung.

**Figure 2.**
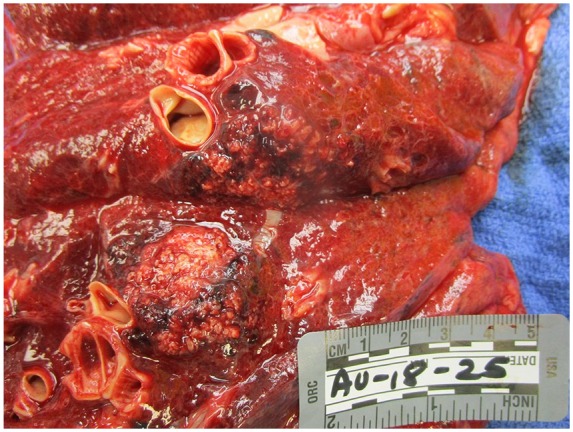
Left lung: left lower lobe tracheobronchial tree is seen, with 2 discrete malignant appearing lung masses.

On gross evaluation of the lungs, the tracheobronchial tree was patent except for the right lower lobe bronchioles, which was filled with thick mucoid material consistent with an infectious process. There were numerous apical blebs and thickened fibrosis diffusely spread throughout the lung parenchyma. The right lung had a large 8.8 × 6.6 cm poorly defined friable mass in the right apex infiltrating the chest wall with rib erosions. The dissected mass was chalky white and fleshy consistent with necrosis (Figure 1). Similarly, the left lung had 3 masses appearing, the largest being 3.1 × 3.0 × 2.2 cm (Figure 2). On dissection of the left upper lobe, we noted abundant amount of necrotic tissue with abscess formation walled by brown pigmented macrophages.

**Figure 3.**
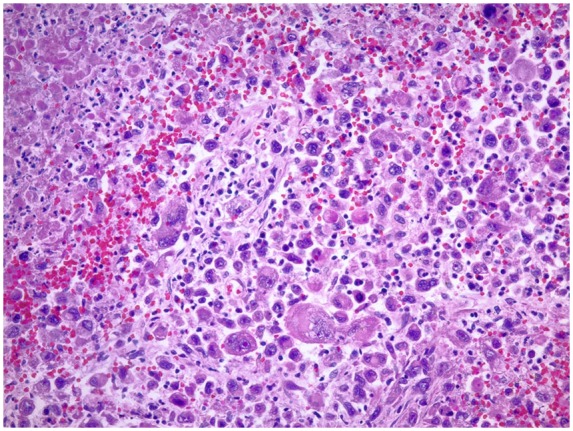
Left lung poorly differentiated large cells with vesicular nuclei, prominent red nucleoli, binucleated to multinucleated bizarrely shaped cells.

On hematoxylin and eosin–stained slides, the tumor cells had poorly differentiated pleomorphic cells with large vesicular nuclei, prominent red nucleoli, and binucleated to multinucleated bizarre shapes (Figure 3). IHC investigations were used to help determine the lineage of the malignant neoplasm.

**Figure 4.**
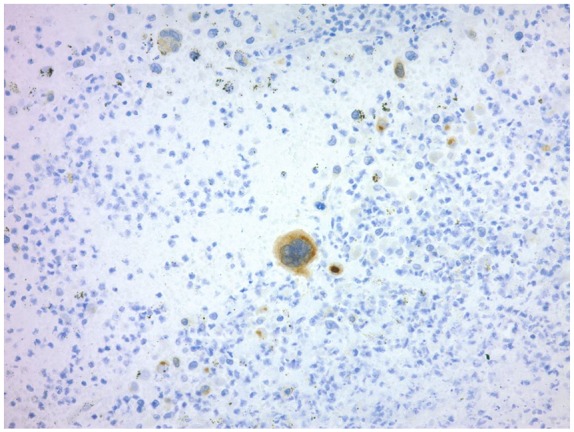
CAM stain: tumor cells showed strongly positive cytoplasmic staining with keratin CAM 5.2.

**Figure 5.**
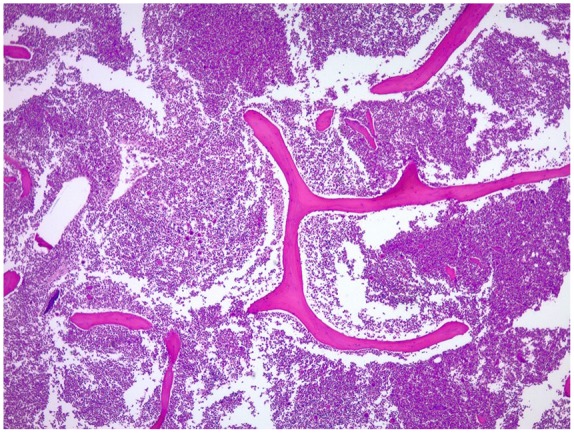
Hypercellular bone marrow consistent with leukamoid reaction.

IHC staining of tumor cells showed strongly positive cytoplasmic staining with keratin CAM 5.2 (Figure 4). Pancytokeratin AE1/3 was negative. Squamous differentiation marker p63 was negative. Glandular differentiation marker thyroid transcription factor 1 was negative. Myogenic differentiation marker desmin was negative. WBC markers CD45 LCA (leukocyte common antigen), CD3, CD20, and CD30 were negative. Histiocytic markers CD68 and CD1a were negative. Dendritic cell markers CD21 and CD23 were negative. Neuroendocrine differentiation marker synaptophysin was negative. Melanocyte differentiation marker HMB-45 was negative. IHC staining for epithelial membrane antigen, ALK-1, human chorionic gonadotropin, and S-100 protein were negative. A periodic acid-Schiff stain was negative for periodic acid-Schiff positive cytoplasmic inclusions. Furthermore, despite prior “dry tap,” the bone marrow was hypercellular with exuberant leukemoid reaction and metastatic large cell neoplasm (Figure 5).

**Figure 6.**
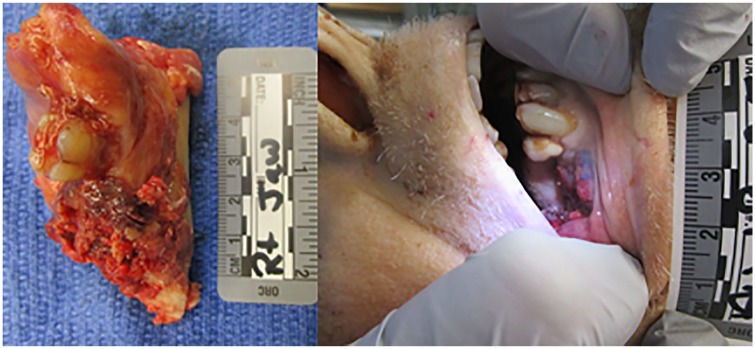
Right mandibular malignant lesion.

Interestingly, the presumed right mandibular abscess identified on CT scan was confirmed to be a large cell neoplasm with strongly necrotic tissue (Figure 6). Despite multiple sites of necrosis blood cultures drawn from central venous line showed negative for microbiological growth. The left lung culture and pericardial fluid showed no microbiologic growth. His right mandible and broth from the right lower lobe of lung grew *Candida albicans*. Cytology from pleural fluid identified malignant appearing cells.

**Figure 7.**
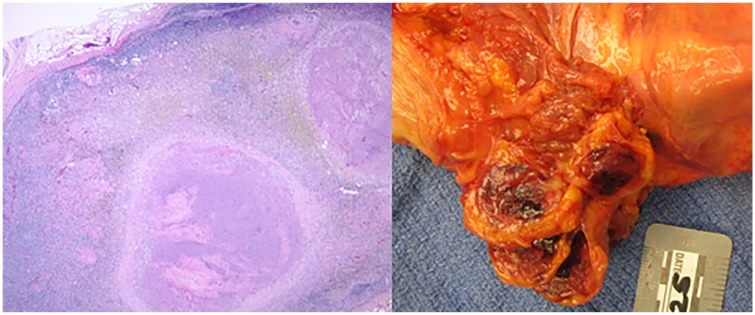
Microscopic and gross imaging of submandibular lymph node consistent with metastatic disease.

**Figure 8.**
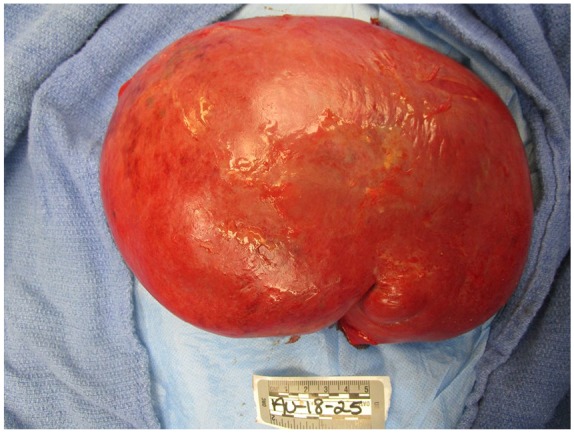
Splenomegaly was noted.

**Figure 9.**
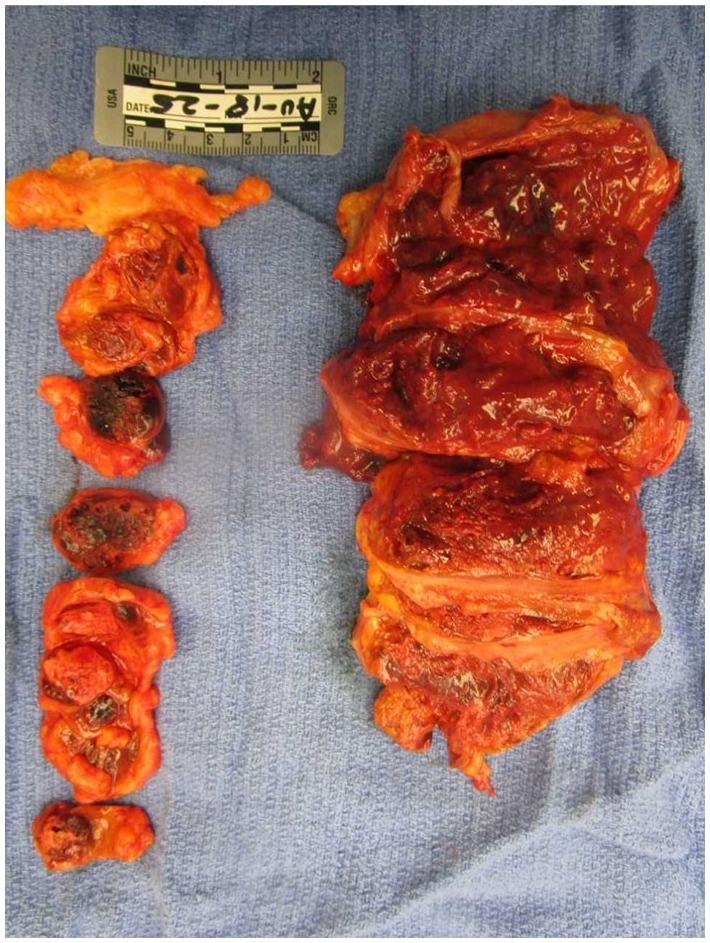
Right (right) and left (left) adrenal glands noted to be pink-brown in color with a cut surface has a thin rim of yellow-brown cortex with a multinodular heterogeneous mass that is white to fleshy to hemorrhagic and necrotic.

Furthermore, the submandibular and mediastinal lymph nodes were dissected. Submandibular and parabronchial lymph nodes on the right were positive for metastatic large cell neoplasm (Figure 7); however, lymph nodes on the left were not consistent with metastatic disease. Four paraaortic lymph nodes from the arch of aorta and 4 abdominal aorta lymph nodes were positive for metastatic large cell neoplasm. Greater mesenteric lymphadenopathy was also noted to be consistent with metastatic large cell neoplasm. The spleen was large with markedly congested parenchyma with rare atypical cells but without infarction (Figure 8). The right adrenal gland was pink-brown with a smooth surface measuring 12.1 × 7.1 × 4.7 cm and weighing 250 g. Left adrenal gland measures 8.1 × 2.5 × 1.5 cm and weighed only 70 g. Both adrenal glands had multiple metastatic nodules, hemorrhage, and diffuse necrosis (Figure 9).

## Discussion

Sarcomatoid carcinoma is a subtype of non–small cell lung cancer. Based on the 2015 World Health Organization classification, it is defined by the presence of a “sarcoma or sarcoma-like component.” They have further divided this into 5 subtypes: spindle cell carcinoma, giant cell carcinoma, pleomorphic carcinoma, carcinosarcoma, and biphasic pulmonary blastoma.^5^ GCCL has been observed more frequently in males than in females,^2,3^ and greater in smokers as compared with nonsmokers. The mean age of diagnosis is at 62.3 years.^3^ Some risk factors associated with sarcomatoid carcinoma of the lung include smoking tobacco products and exposure to asbestos in building construction and electrical insulation.^6^ Pan-cytokeratin (CAM 5.2 and LP 34) has been reported to be present in sarcomatoid carcinoma of the lung.^7^ Staining with CK7 and CK20 antibodies can help discriminate between primary lung adenocarcinoma and adenocarcinoma from a different organ that has metastasized to the lung. Also, CAM 5.2 positive in the absence of other stains strengthened our final diagnosis of sarcomatoid carcinoma of the lung.

This is a unique case provided that autopsy was performed and helped provide a greater insight to the degree of organ involvement in this rare disease. Few reports include such extensive postmortem histologic and anatomic images. Data are lacking regarding treatment options. It has been previously described that surgery can be an adequate approach for treatment in localized cases. However, there appears to be no overall survival benefit in the type of chemotherapy used in advanced cases of sarcomatoid lung cancer.^8^ Our patient suffered from advanced disease and expired prior to having an established diagnosis, and thus did not receive oncologic therapy. However, considering his advanced metastatic disease, it is unlikely that any oncologic treatment may have been promising. He was treated for sepsis and post-obstructive pneumonia with broad spectrum antibiotics, fluid resuscitation, blood products, and supportive therapy.

Finally, this study sheds light on this rare disorder by providing postmortem images that are not often accessible, and provides a pathological and IHC review of these stains, which led to our diagnosis.
